# Tumour microenvironment in pheochromocytoma and paraganglioma

**DOI:** 10.3389/fendo.2023.1137456

**Published:** 2023-03-22

**Authors:** Serena Martinelli, Francesca Amore, Letizia Canu, Mario Maggi, Elena Rapizzi

**Affiliations:** ^1^Department of Experimental and Clinical Biomedical Sciences “Mario Serio”, University of Florence, Florence, Italy; ^2^Centro di Ricerca e Innovazione sulle Patologie Surrenaliche, Azienda Ospedaliera Universitaria (AOU) Careggi, Florence, Italy; ^3^European Network for the Study of Adrenal Tumours (ENS@T) Center of Excellence, Florence, Italy; ^4^Department of Experimental and Clinical Medicine, University of Florence, Florence, Italy

**Keywords:** tumour microenvironment, pheochromocytoma/paraganglioma, cancer associated fibroblasts, extracellular matrix, catecholamines

## Abstract

Pheochromocytomas and Paragangliomas (Pheo/PGL) are rare catecholamine-producing tumours derived from adrenal medulla or from the extra-adrenal paraganglia respectively. Around 10–15% of Pheo/PGL develop metastatic forms and have a poor prognosis with a 37% of mortality rate at 5 years. These tumours have a strong genetic determinism, and the presence of succinate dehydrogenase B (SDHB) mutations are highly associated with metastatic forms. To date, no effective treatment is present for metastatic forms. In addition to cancer cells, the tumour microenvironment (TME) is also composed of non-neoplastic cells and non-cellular components, which are essential for tumour initiation and progression in multiple cancers, including Pheo/PGL. This review, for the first time, provides an overview of the roles of TME cells such as cancer-associated fibroblasts (CAFs) and tumour-associated macrophages (TAMs) on Pheo/PGL growth and progression. Moreover, the functions of the non-cellular components of the TME, among which the most representatives are growth factors, extracellular vesicles and extracellular matrix (ECM) are explored. The importance of succinate as an oncometabolite is emerging and since Pheo/PGL SDH mutated accumulate high levels of succinate, the role of succinate and of its receptor (SUCNR1) in the modulation of the carcinogenesis process is also analysed. Further understanding of the mechanism behind the complicated effects of TME on Pheo/PGL growth and spread could suggest novel therapeutic targets for further clinical treatments.

## Introduction

Pheochromocytomas (Pheo) and paragangliomas (PGL) are rare neuroendocrine tumours of chromaffin cells originating from the ectodermal portion of the neural crest (1, 2). Pheo originates from the adrenal medulla while PGL derives from extra-adrenal sympathergic paraganglias. These tumours are commonly referred as Pheo/PGL (3), and they usually secrete catecholamines leading to hypertension and myocardial degenerative effects (4).

Approximately 80% of Pheo/PGL are related to mutations in one out of more than 20 genes (5–7), these different mutations can be separated into three main clusters (**Table 1**). The pseudohypoxic signalling cluster (cluster-1) is related to mutations of genes encoding for proteins that are associated with significant regulation of the hypoxia signalling pathway; these include mutations in genes encoding for HIF2α (hypoxia-inducible factor-2α), Krebs cycle enzymes such as succinate dehydrogenase subunits [SDHx (SDHA, SDHB, SDHC, SDHD)], fumarate hydratase (FH), malate dehydrogenase 2 (MDH2), and isocitrate dehydrogenase 1 (IDH1). Moreover, this cluster includes mutations in succinate dehydrogenase complex assembly factor-2 (SDHAF2), von Hippel–Lindau tumour suppressor (*VHL*) and egl-9 prolyl hydroxylase-1 and -2 (*EGLN1/2*) genes. All these mutations promote HIFα stabilization and accumulation resulting in increased angiogenesis *via* changes in vascular endothelial growth factor-1 and -2 receptors (VEGFR1/2) and platelet-derived growth factor-β receptor (PDGFR) transcription. The kinase signalling cluster (cluster-2) is related to mutations of genes encoding for proteins that belong to the phosphatidylinositol-3-kinase (PI3K)/mammalian target of rapamycin (mTORC1) pathway/receptor kinase signalling and comprises mutations in the rearranged-during-transfection (*RET*) proto-oncogene, neurofibromin 1 (*NF1*) tumour suppressor, *H-RAS* and *K-RAS* proto-oncogenes, transmembrane protein 127 (*TMEM127*), and Myc-associated factor X (*MAX*). Most recently, the Wnt signalling cluster (cluster-3) has been described as being of pathological significance. Tumours mutated for the Cold Shock Domain-containing E1 (*CSDE1*) and the Mastermind Like Transcriptional Coactivator 3 (*MAML3*) fusion genes belong to cluster 3 (38, 39). Among the genes encoding for the *SDH* subunits, *SDHB* was found to be highly related to metastatic forms (40). Since it is impossible to differentiate non-metastatic and metastatic Pheo/PGL based upon clinical or even histopathological findings, all Pheo/PGL are currently considered potentially metastatic tumours (WHO 2017 classification) (41). As a result, all patients with Pheo/PGL require long and intensive follow up.

**Table 1:** Pheo/PGL susceptibility genes.

Gene	Cluster	Mutation status	Discovery year
***VHL***	I	Germline/somatic	Latif F. et al. (8)
***SDHD***	I	Germline/somatic	Baysal B.E. et al. (9)
***SDHC***	I	Germline/somatic	Niemann S. et al. (10)
***SDHB***	I	Germline/somatic	Astuti D. et al. (11)
***SDHAF2***	I	Germline/somatic	Hao H.X. et al. (12)
***SDHA***	I	Germline/somatic	Burnichon N. et al. (13)
***IDH1***	I	Somatic	Gaal J. et al. (14)
***IDH2***	I	Somatic	Yao L. et al. (15)
***HIF2A/EPAS1***	I	Somatic	Lorenzo F.R. et al. (16)
***FH***	I	Germline/somatic	Castro-Vega LJ et al. (17)
***PHD1/EGLN2***	I	Germline	Yang C. et al. (18)
***PHD2/EGLN1***	I	Germline	Yang C. et al. (18)
***H3F3A***	I	Somatic	Toledo RA et al. (19)
***GOT2***	I	Germline/somatic	Remacha L. et al. (20)
***IDH3B***	I	Germline	Remacha et al. (20)
***KIF1B***	I	Germline/somatic	Evenepoel L et al. (21)
***DNMT3A***	I	Germline/somatic	Remacha et al. (22)
***SLA25A11***	I	Germline/somatic	Buffet A. et al. (23)
***MDH2***	I	Germline	Calsina B. et al. (24)
***DLST***	I	Germline/somatic	Remacha L. et al. (25)
***SUCLG2***	I	Germline/somatic	Hadrava Vanova et al. (26)
***RET***	II	Germline/somatic	Santoro M. et al. (27)
***NF1***	II	Germline/somatic	Xu W. et al. (28)
***MEN1***	II	Germline/somatic	Schussheim et al. (29)
***PTEN***	II	Somatic	Van Nederveen FH et al. (30)
***TMEM17***	II	Germline/somatic	Qin Y. et al. (31)
***MAX***	II	Germline/somatic	Comino-Mendez I. et al. (32)
***CDK2B***	II	Somatic	Muscarella P. et al. (33)
***KRAS***	II	Somatic	Hrascan R. et al. (34)
***HRAS***	II	Somatic	Crona J. et al. (35)
***BRAF***	II	Somatic	Lucchetti A. et al. (36)
***ATRX***	II	Somatic	Fishbein L. et al. (37)
***FGFR1***	II	Somatic	Toledo RA. et al. (19)
***MET***	II	Germline/somatic	Toledo RA. et al. (19)
***MERKT***	II	Germline/somatic	Toledo RA. et al. (19)
***CSDE1***	III	Somatic	Fishbein L. et al. (5)
***UBTF-MAML3***	III	Somatic	Fishbein L. et al. (5)

Tumorigenesis is a multiphase process dependent on several modifications at cellular and tissue levels, leading to sustain proliferative signalling, evasion from growth suppressors and from cell death, replicative immortality, and induction of angiogenesis, invasion, and metastasis (42, 43). Beyond genetic alterations, the interplay among cancer cells and tumour microenvironment (TME) components has a central role in tumour initiation and progression (44). Indeed, tumours are characterized by high cellular heterogeneity, which includes cancerous cells, non-cancerous cells, and non-cellular components, giving origin to TME (45). Since TME has been reported to have a central role in fostering many human malignancies (46–48), it has been recently proposed as a potential target for tumour therapy (49, 50).

Pheo/PGL current treatments include surgery, systemic therapies, and radioiodine, none of which are effective for metastatic forms, and only a limited number of clinical trials are in progress evaluating targeted therapies for the different Pheo and PGL subtypes (51, 52). Therefore, it is essential to elucidate novel molecular targets, including TME, to diversify and improve available therapies.

## Cellular components of Pheo/PGL TME

**Figure 1:**
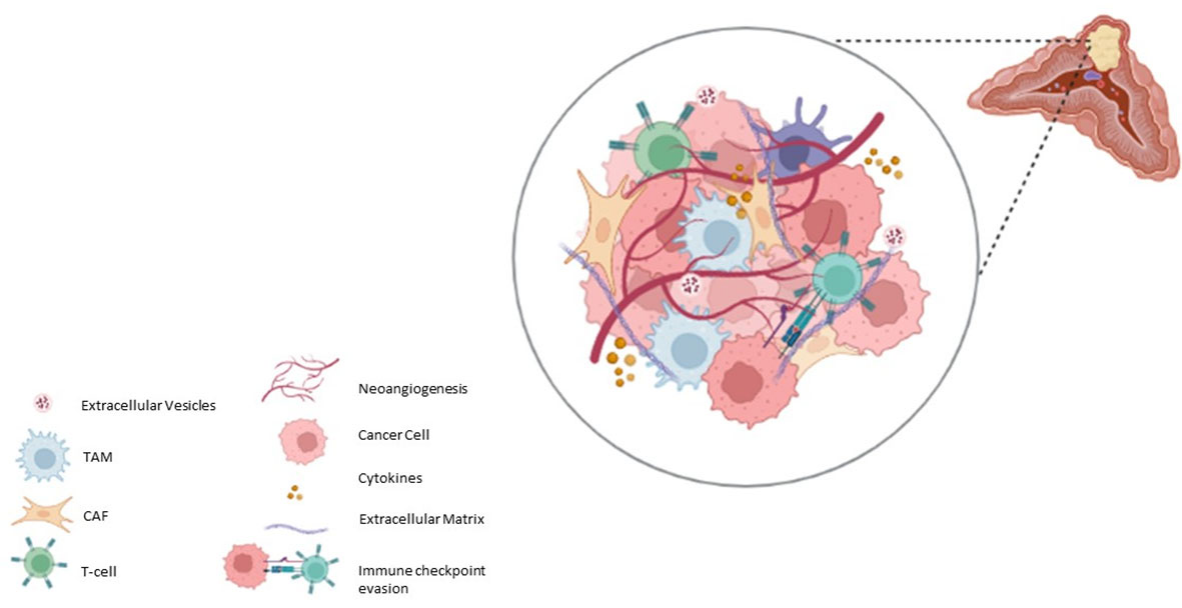
Schematic representation of Pheo/PGL microenvironment.

Pheo/PGL tumours can contain small numbers of ganglion cells, neuroblasts, melanin-containing cells, and sustentacular cells (53–55). Other cell components of TME have been reported in Pheo/PGL, such as great number of fibroblasts, endothelial cells, macrophages as well as immune cells (56) (**Figure 1**). In the next paragraphs we will talk about the roles of the most important cellular actors of the TME.

## Cancer associated fibroblasts

In physiological conditions, fibroblasts are cellular component of tissues, and they are involved in providing structural scaffolding and trophic ancillary function for the cells of the tissues. Within the tumours, cytokines released by cancer cells convert fibroblasts into a permanently activated myofibroblast-like form, called cancer associated fibroblasts (CAFs) (57). This chronic activation of fibroblasts within TME is crucial for cancer progression. It has been reported that CAFs have an increased glycolysis and produce high-energy nutrients that facilitate biogenesis in malignant cells, a process referred to as the “reverse Warburg effect”. The Reverse Warburg Effect describes when glycolysis in the cancer-associated stroma metabolically supports adjacent cancer cells. This catabolite transfer allows cancer cells to generate ATP, increase proliferation, and reduce cell death (58, 59). We have previously demonstrated that microenvironment, represented by fibroblasts, strongly affects neuroblastoma metabolism and growth capacity. In particular, we showed that primary fibroblasts and tumour cells establish reciprocal metabolic changes. Tumour cells co-cultured with human fibroblasts, showed a significant decrease in glucose uptake, an increase in lactate uptake, and a 92% increase in proliferation rate compared with single-cultured counterpart (60). Moreover, murine primary fibroblasts co-cultured with mouse pheochromocytoma cells (i.e. mouse tumour tissue-derived cells, MTT) increased glucose uptake and produced lactate, thus shifting to a Warburg-like glycolytic metabolism. Lactate was then released by fibroblasts, and uploaded by tumour cells, which in turn increased anabolic processes, proliferation, and metalloproteinase activation (60). We next demonstrated that CAFs induced an extraordinary increase of the migration/invasion of MTT cells (61). However, modulating the concentration of nutrients, in particular lowering glucose concentration, CAFs undergo a metabolic impairment, and they are not any longer able to induce cancer cell invasion (62). Fernandez and colleagues demonstrated that overexpression of insulin-like growth factor 1 receptor type 1 (IGF1R) was associated with high risk of metastasis in patients with familial Pheo and PGL, through stimulating survival and anchorage independent growth *in vitro*. In the same work, they also demonstrated that circulating insulin-like growth factor 1 (IGF1) had a critical role in maintaining tumour phenotype and survival of already transformed Pheo cells *in vivo* (63). Another study conducted by the same group using the mouse pheochromocytoma cell line (MPC) demonstrated that IGF1R deficiency in fibroblasts had effects on the survival of Pheo cells before tumour establishment (64). In fact, a decreased production of fibronectin, IGF1 and IGFBP2 by haploinsufficient IGF1R fibroblasts, together with a downregulation of integrins expression in tumour cells, impaired the survival of tumour cells (65). Their results suggest that IGF1 through IGF1R may be involved in early stages of tumour establishment, contributing to tumour cells anchorage by interaction with soluble and non-soluble factors produced by CAFs.

## Tumour associated macrophages

Macrophages are innate immune cells pivotal for tissue homeostasis, removal of superfluous cells, and inflammatory responses to infections (66, 67). In response to tumour microenvironment signals, macrophages undergo phenotype shift between M1 or M2. In particular, M1 macrophages are regarded as anti-tumor and typically identified by the surface markers CD86 and CD64, while M2 are polarized macrophages, commonly considered tumor-associated macrophages (TAMs) and typically express the surface markers CD206 and CD163. A large number of studies suggests that TAMs serve as prominent metastasis promoters in the TME, which orchestrate almost all of the steps of tumour metastasis (68). In cancers, high macrophage infiltration often associates with a poor prognosis or with tumour progression in many types of solid tumours, including breast (69), bladder (70), head and neck (71), glioma (72), melanoma (73), and prostate cancer (74).

These macrophages are associated with wound healing and tissue repair. Macrophages have not been reported as a major component of Pheo/PGL, since recently when Farhat and colleagues identified, in sections of Pheo/PGL, a dense population of cells positive for both CD163, a highly specific M2-type TAM marker, and CD68 involved in phagocytic activity of macrophages (75). In this work, three out of four Pheo/PGL tumours with mutations in genes encoding for the SDH subunits showed the highest levels of CD163 compared with tumours harbouring different mutations. Very recently, Tufton and colleagues confirmed the presence of macrophages, lymphocytes and neutrophils by immunohistochemistry on Pheo/PGL tumour samples (76). They not only found a higher proportion of immune cells with a predominance of macrophages, in tumour tissue compared with non-neoplastic adrenal medulla tissue, but also a higher proportion of M2:M1 macrophages and T-helper lymphocytes in aggressive tumours compared with indolent ones. Also, Ghosal and colleagues studied the prognostic immune cell infiltration signatures in neuroendocrine neoplasms (NENs), particularly Pheo/PGL, by analysing tumour transcriptomic data from The Cancer Genome Atlas (TCGA) and other published tumour transcriptomic data of NENs. The authors correlated immune cell infiltration patterns with known Pheo/PGL molecular subtypes that carry the risk of aggressive and metastatic phenotypes in this disease. By further analysing the immune signatures in other tumours such as gastroenteropancreatic neuroendocrine tumours (GEPNETs), and small cell lung carcinomas (SCLCs), they associated similar immune signatures with metastatic phenotypes and calculated patient prognosis among various NENs and emphasizing Pheo/PGL. These results identify an immune infiltration signature for metastatic Pheo/PGL and NENs (77). These data showed that Pheo/PGL are immunologically active tumours, and immunotherapy might be considered as a potential treatment for patients with metastatic Pheo/PGL.

**Figure 2:**
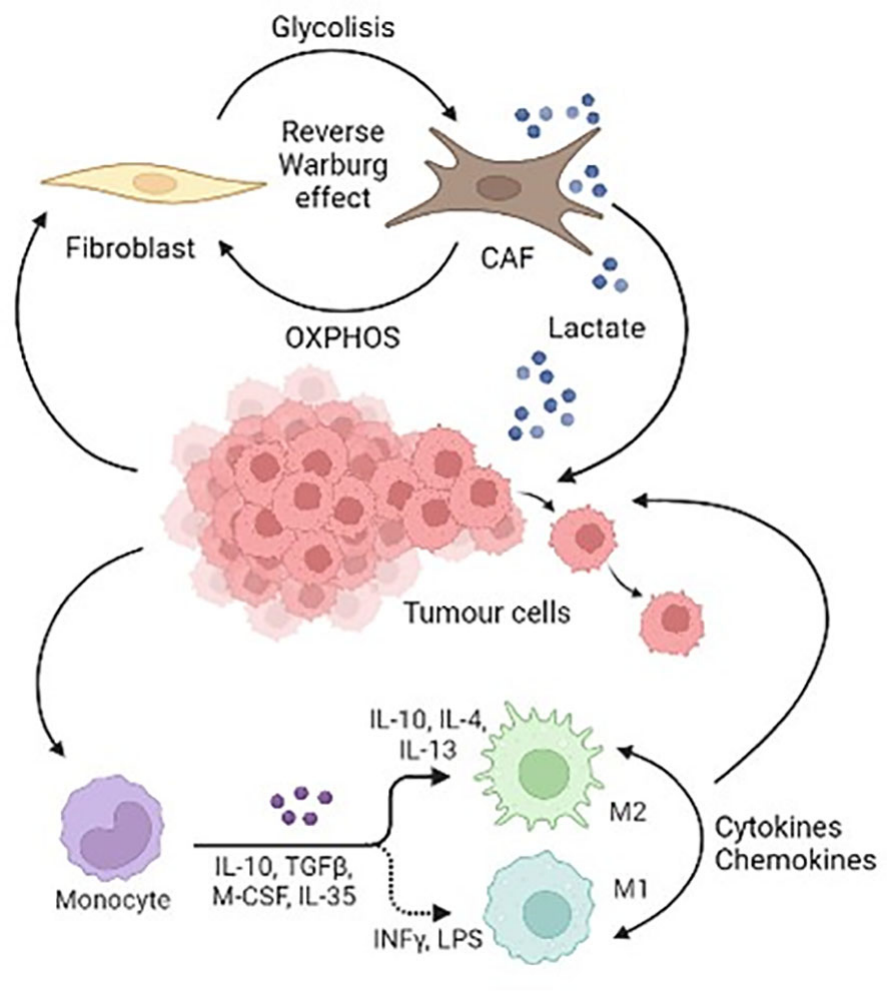
Schematic representation of the cellular components of Pheo/PGL tumour microenvironment.

**Figure 2** is a schematic representation of the cellular components of Pheo/PGL TME.

## Non-cellular components of Pheo/PGL TME

In addition to cellular components, there are many pro-tumorigenic non-cellular elements that play important roles in tumour maintenance and progression. The autocrine and the paracrine signalling between TME and cancer cells leads to the production and remodelling of the extracellular matrix (ECM), induces the production of extracellular vesicles (EVs), the secretion of growth factors, cytokines, chemokines, and metabolites, and stimulates blood and lymph vessel networks formation (78). In addition to the crosstalk between the diverse cells of the TME, the situation of the TME is even more complicated because of the interaction between cellular and the non-cellular components, such as pH conditions, hypoxia, or soluble factors which could indeed change the conditions of TME that in turn support the tumour progression and metabolism (79).

## Extracellular matrix

The extracellular matrix (ECM) is a highly dynamic structure that is present in all tissues and continuously undergoes controlled remodelling. The ECM interacts with cells to regulate diverse functions, including proliferation, migration and differentiation (80). ECM is composed by fibrillar collagens, fibronectin, elastin, keratins and laminins (81, 82). In addition, some cancers, are particularly rich in hyaluronan (83).

Recent evidence has claimed that changes in the deposition, composition, organization, and even post-translational modification of ECM have a key role in tumour progression. Both cancer and stromal cells contribute to deposition of ECM, and its properties alter tumour features, such as the potential to form metastasis (84). Cells modify the ECM not only by producing ECM components, but also by secreting enzymes which modify the ECM, such as transglutaminases (TGMs) and lysyl oxidases (LOXs), which crosslink ECM components (85, 86), MMPs (metalloproteinases), ADAMs (a disintegrin and metalloproteinases), and ADAMTSs (a disintegrin and metalloproteinase with thrombospondin motifs) that proteolytically degrade ECM components (87, 88). The dysregulated activity of these enzymes, together with the excessive deposition of ECM components and their reduced turnover, are typical of malignant lesions. Tumour ECM is different from physiological ECM (89, 90); for example, the tumour ECM is usually stiffer than the physiological one: this plays a key role in maintaining CAF phenotype, enhancing cancer and stromal cell invasion, cancer cell-endothelium interactions, epithelial to mesenchymal transition (EMT), and immune cell recruitment (91–93). Targeting ECM production is also a potential therapeutic strategy because the composition and mechanical properties of the ECM are established active drivers of tumour pathology (94, 95). Regarding human Pheo/PGLs, transcriptome and methylome data revealed that high levels of promoter methylation of of Keratin19 (KRT19) were able to distinguish SDHB-mutated tumours from all other Pheo/PGL tumours (96, 97). In a cell line model of Pheo/PGL (imCC), the knock down of *SDHB* leaded to a global hypermethylation and significative downregulation of KRT19, supporting the functional role of the epigenetically silenced *Krt19* gene. *Krt19* repression participates to some phenotypic modifications such as increase in migration capacity and epithelial to mesenchymal transition (EMT) induction, observed in SDHB-deficient cells and thus in SDHB associated metastatic phenotype (96). Another work described some prognostic genes related to TME in Pheo/PGLs, based on weighted gene co-expression network analysis (98) as in the algorithm proposed by Yoshihara et al. (99). A comprehensive bioinformatic analysis was performed, and the authors identified three TME related genes: ADGRE1 (Adhesion G Protein-Coupled Receptor E1) involved in cell adhesion to the ECM, CCL18 (C-C Motif Chemokine Ligand 18) a chemokine produced mainly by antigen-presenting cells, and LILRA6 (Leukocyte Immunoglobulin Like Receptor A6) a membrane protein. ADGRE1 was associated with longer overall survival in Pheo/PGLs which indicated a protective role in Pheo/PGLs biogenesis. However, the mechanism of ADGRE1 in Pheo/PGLs development remains unknown and further research is required to be investigated (98).

## Extracellular vesicles

Extracellular vesicles (EVs) are phospholipid-bilayer enclosed vesicles known as important mediators of intercellular communication. EVs transfer biologically active molecules and genetic material (mRNA, microRNA, siRNA, DNA, protein and lipid) through paracrine mechanisms, which associate the regulation of inflammation, disease development and progression, pre-metastatic niche formation, and the metastatic organotropism of different tumour types (100). EVs are secreted from all type of cells in physiological and pathological conditions and higher amount of EVs have been evaluated from cancerous cells (101). EVs are a family comprising three main members that include exosomes (ca. 30–150 nm), microvesicles (ca. 50 nm–1 mm) and apoptotic bodies. They can be distinguished by their triggering mechanisms and biophysiological properties.

Exosomes (ca. 30–150 nm) result from the release of multivesicular bodies present inside the cellular endosomal system and carry various signalling in the locally pathways and distant target cell *via* transmitting heterogeneous cargoes. A high number of exosomes were demonstrated in cancer patients which are able to promote metastatic progression by inflammation, proliferation and suppressing of immune system as a means of cancer immune evasion (102). Xie and colleagues studied the effects of adipose mesenchymal stem cell-derived exosomes (ADSC-exo) on PC12 rat adrenal pheochromocytoma cell line and they found that ADSC-exo significantly promoted PC12 cell proliferation in an exosome dose-dependent manner. Moreover, ADSC-exo also enhanced PC12 cell migration through activation of the PI3K/AKT signalling pathway (103). Finally, exosomes are good biomarker candidates for non-invasive diagnosis since they contain RNA, DNA, and proteins (39). However, the presence of DNA in exosomes is usually dependent on cell type, and the ability of the exosomal DNA to reflect the mutational status of the cells of the tumour of origin in Pheo/PGL patients is largely unknown. Thus, Wang et al. focused their attention on exosomal DNA from Pheo/PGL exosomes, and they hypothesized that human serum exosomes may contain information regarding the presence of mutations of RET, VHL, HIF2α, and SDHB reflecting the mutation of their parental cells located in the tumour of origin. To assess this, they analysed samples from 12 Pheo or PGL patients whose somatic tumour mutations was already identified by genetic diagnosis. This study first revealed that Pheo and PGL exosomes contain double stranded DNA (dsDNA) that can reflect the mutation status of susceptibility genes and cover nearly all chromosomes (104). This suggests the use of exosomes as non-invasive genetic markers in one of the most effective somatic mutation screens for the genetic diagnosis and preoperative assessment of Pheo and PGL (104). Another interesting feature in the use of exosomes is their capacity for carrying a payload of proteins or nucleic acids to target cells that may be effective in developing novel cancer therapies that are less harmful than chemotherapy. For most drugs, only a relatively small amount reaches the lesion to exert a therapeutic effect. This reduces the efficacy and can cause toxicity and adverse side effects to the patient. Moreover, exosomes have many advantages, such as small size, natural molecular transport properties, and good biocompatibility (105). Tumour therapy based on exosomes may become an important part of personalized medicine, because they can be loaded with different types of compounds, such as small-molecule chemical drugs, proteins and nucleic acids (106).

## Vascular endothelial growth factor

Tumour development and its survival depends on an adequate supply of oxygen and nutrients (107). Angiogenesis, the development of new blood vessels from established vasculature, provides growth and hematogenous dissemination of the cancer cells (108, 109). Several pro-angiogenic and anti-angiogenic molecules are involved in the regulation of this process (109). Among them, vascular endothelial growth factor (VEGF; VEGF-A) is the most well-characterized angiogenic factor (110). VEGF-A, a cytokine that exerts a critical role in both pathologic and physiologic angiogenesis, binds and activates two tyrosine kinase receptors: vascular endothelial growth factor receptor 1 (VEGFR-1; Flt-1) and vascular endothelial growth factor receptor 2 (VEGFR-2; KDR; Flk-1) (111). On binding to its receptors, VEGF-A initiates a cascade of signalling events resulting in the activation of downstream proteins, including mitogen-activated protein kinase (MAPK) and phosphatidylinositol 3-kinase (PI3K) pathways (112, 113). Several studies have demonstrated that VEGF-A mRNA is upregulated in different human tumours, including prostate (114), lung (115), gastrointestinal tract (116) and kidney (117). Furthermore, VEGF-A expression has been associated with poor prognosis in human tumours (101, 118).

Pheo/PGL are well-vascularized tumours, but the role of VEGF-A and its receptors is poorly understood. Takekoshi et al. (119) observed increased levels of VEGF-A and its receptors in 11 tumour specimens of Pheo and suggested that upregulation of these molecules may be important in Pheo pathogenesis (119). Moreover, associations between the increased intensity of VEGF-A expression and micro vessel density (MVD) in Pheo tissue and metastatic phenotype have been reported (120). Other studies simply related the increased in VEGF-A expression with malignancy, without finding an association with MVD (120–123). Ferreira et al. (124) assessed VEGFR-1 and VEGFR-2 as markers of angiogenesis in samples of hereditary or sporadic Pheo. VEGF-A and VEGFR-1 staining were detected in all Pheo tissue samples analysed, whereas VEGFR-2 expression was present in approximately 80% of the cases. Moreover, VEGF-A and its receptors were up-regulated in metastatic Pheo, suggesting that these molecules might be considered as therapeutic targets for unresectable or metastatic tumours (124).

## Catecholamines

The adrenomedullary chromaffin cells are embryologically derived from migrating neural crest cells that develop into sympathoadrenal progenitors (125, 126). These sympathoadrenal progenitor cells also give rise to the chromaffin cells present in the sympathetic chain and prevertebral paraganglia. The principal function of the adrenal medulla is the biosynthesis and the secretion into the circulation of the catecholamine epinephrine (127). The measurement of plasma or urinary metanephrines (metanephrine and normetanephrine) and methoxytyramine, which are the O-methylated metabolites of catecholamines and dopamine, respectively, are strongly recommended for initial screening of Pheo/PGL and in the follow up (128–130).

Preclinical data have shown that neurotransmitters released in peripheral tissues from nerve endings may influence carcinogenesis, affect the tumour microenvironment, and directly potentiate both proliferation and migration of cancer cells (42). *In vitro* studies showed that administration of either agonists or antagonists of adrenergic β-receptors might significantly affect the proliferation and migration of cancer cells (131). For example, it has been found that norepinephrine significantly potentiates the proliferation of cancer cells, and this effect can by blocked by administration of β-blockers (132, 133). Additionally, several clinical studies determined that there is an effect of β-blockers on both reducing cancer progression and increasing the survival of oncological patients (134). If the inhibition of β2-adrenergic receptors reduces cancer progression, it can be hypothesized that overactivation of these receptors may induce the opposite effect. In patients with Pheo, plasma catecholamine levels are enormously increased (135). These catecholamines may profoundly stimulate β2-adrenergic receptors and therefore pheochromocytoma may represent a clinical model of exaggerated sympathetic nervous system activity. Moreover, higher levels of dopamine and its metabolite, methoxytyramine, are reported to be associated with a heightened risk of recurrence together with other factors, such as hereditary predisposition, younger age at initial tumour diagnosis, extra-adrenal tumour location, larger tumour size (136–138). Adrenal glucocorticoid produced by adrenal cortex stimulates synthesis and activity of phenylethanolamine N-methyltransferase (PNMT), which is the enzyme that convert norepinephrine to epinephrine in the adrenal medulla. Because exogenous glucocorticoid supplementation suppresses endogenous glucocorticoid synthesis by negative feedback at the level of the hypothalamus and pituitary, some groups had shown that this inhibits the synthesis and activity of PNMT *in vitro* (139) and *in vivo* (140) in physiologic conditions. Sharara-Chami and colleagues studied whether exogenous glucocorticoid could also inhibit the rise in epinephrine synthesis in the setting of basal or stress-induced mouse model (141). The results of this study showed that in mice without stress, when adrenocorticotropic hormone is low, high doses of exogenous dexamethasone stimulate PNMT and catecholamine synthesis, likely independently of adrenal corticosterone concentration. After stress, adrenocorticotropic hormone levels are elevated, and exogenous dexamethasone suppresses endogenous corticosterone and PNMT production. Nonetheless, catecholamines increase, possibly due to direct neural stimulation, which may override the hormonal regulation of epinephrine synthesis during stress (141). Catecholamines signal primarily through the β2-adrenergic receptors present on innate and adaptive immune cells which are critical in responding to infections caused by pathogens. In general, this adrenergic input, particularly chronic stimulation, suppresses lymphocytes and allows infections to progress: β-adrenergic signalling protects tumour cells from T cell surveillance *via* the suppression of MHC-I expression and the upregulation of PD-L1. These findings highlight that β-adrenergic signalling antagonism might be a beneficial strategy for cancer therapy (142).

## Succinate

Succinate is a molecule formed from succinyl-CoA synthetase and converted by succinate dehydrogenase (SDH) to fumarate in the Krebs Cycle. Pheo/PGLs that harbour a SDHx mutation are characterized by dysfunction of the SDH enzyme. As the conversion to fumarate is impaired, a substantial accumulation of succinate occurs. The accumulation of succinate is shuttled from the mitochondrial matrix to reach the cytoplasm where it mediates different oncogenic effects such as the inhibition of prolyl hydroxylase (PHD), which is responsible for hydroxylation of HIF1α. Blocking PHD prevents HIF1α degradation and induces expression of several HIF-target genes that are known to be involved in angiogenesis (143). Pollard and colleagues described the same phenomenon in SDH- and fumarate hydratase (FH)-mutated Pheo/PGLs (144), that indeed, are characterized by 25-fold higher succinate levels than tumours mutated in the other susceptibility genes (145). Alongside PHD inhibition, accumulation of succinate inhibits jumonji-domain histone demethylases (JmjC) and the ten-eleven translocation (TET) family of DNA methylase (146, 147). This leads to hypermethylation of promotor regions (CpG islands) of several genes involved in tumorigenesis (148, 149). In Pheo/PGLs, SDHx mutations were shown to promote a massive hypermethylation phenotype.

In addition to its role as an oncometabolite, succinate can also act as a ligand for the G protein-coupled receptor 1 (SUCNR1) (150), which has been shown to be expressed in many tissues (151–154). Depending on cell type, this receptor could be couple to different G-proteins, so the effect of its stimulation involves different mechanisms (155). In recent years, several studies have highlighted the role of succinate and SUCNR1 in tumorigenesis (156–158). Moreover, succinate treatment as well as SDHB-silencing has been shown to induce SUCNR1 mRNA and protein expression in human hepatoma cells (159), suggesting a positive feedback of inappropriate succinate accumulation on expression of this receptor. Recently, Matlac and colleagues showed that mRNA expression of SUCNR1 was higher in SDHx mutated Pheo/PGLs compared to cluster 2 tumours. Moreover, they confirmed elevated SUCNR1 protein expression levels in SDHB mutated Pheo/PGLs compared to VHL (Von Hippel-Lindau) mutated Pheo (160). However, little is known about the effects of succinate and its receptor on TME cells. Only very recently, it has been demonstrated that lung cancer-derived succinate, released into the TME, induces macrophage polarization and cancer metastasis by activating SUCNR1 (161). In dendritic cells, succinate enhances the capacity to act as antigen-presenting cells, to migrate towards draining lymph nodes and to produce cytokine in synergy with Toll-like receptor ligands (152, 162, 163). In this scenario, the importance of studying the effects of succinate and SUCNR1 not only in cancer cells, but also in TME, is highlighted. In this view, Pheo/PGL tumour cells with SDHx mutations might represent the best models for these studies.

**Figure 3:**
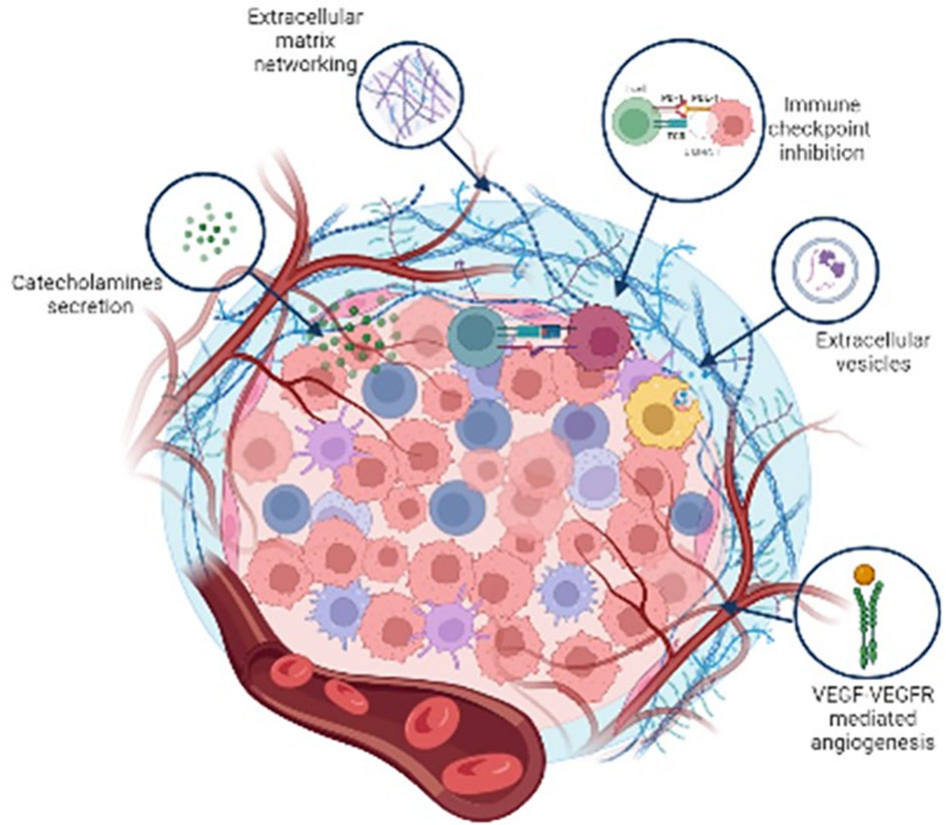
Schematic representation of the major non cellular components of Pheo/PGL tumour microenvironment.

**Figure 3** is a schematic representation of the non cellular components of Pheo/PGL TME.

## Immune phenotype of Pheo/PGL

Inhibition of the programmed cell death (PD-1) receptor-ligand immune checkpoint has recently revolutionised the systemic treatment of malignancies (164). By antagonising the immune-suppressive interaction between PD-1, a T-cell co-inhibitory receptor, and its ligand PD-L1, therapeutic antibodies against this pathway can restore an efficacious anti-tumour immune response, manifest as durable clinical responses in a proportion of patients affected by several cancers such as melanoma, non-small-cell-lung cancer, ovarian cancer and renal cell carcinomas (165). PD-L1 expression by immunohistochemistry has been considered a putative correlated predictor of response to anti-PD-1 therapies and used as a biomarker for the use of immune checkpoint inhibitor therapies (166, 167). Since elevated PD-L1 expression in tumour cells or TME cells is a result of various molecular events including hypoxia (168), and since activation of hypoxia inducible factor (HIF) is a key molecular hallmark in the metastatic progression of Pheo/PGL (169, 170), it has been hypothesized that activation of the hypoxic response might promote cancer-specific immune-tolerance through expression of PD ligands and thus facilitate malignant progression in Pheo/PGL (171). Pinato and colleagues documented for the first time, differential regulation of PD ligands in Pheo/PGLs, where half of the malignant cases expressed at least one of the PD ligands, supporting their potential contribution in shaping the immune-tolerogenic environment (171). Hsu et al. detected PD-L1 expression also in mediastinic PGLs, confirming that PD-L1 expression might be associated with a more aggressive disease in paragangliomas (172).

The CTLA-4 (cytotoxic T-lymphocyte-associated protein 4, CD152) pathway is another commonly targeted pathway in cancer immunotherapy (173). Dum and colleagues (174) identified and quantified lymphocyte subpopulations in several tumours including Pheo/PGLs by staining CTLA-4 which is an inhibitory immune checkpoint receptor and a negative regulator of anti-tumour T-cell function which could be another promising target for immunotherapy.

The metastatic behaviour of Pheo/PGLs before the development of metastasis is poorly understood: if the risk could be predicted before that metastasis occurs, patients would get optimal therapeutic time. To this end, Guo et al. studied PD-L1 expression in Pheo/PGLs and analysed the relationship of PD-L1 expression and malignant behaviour before distant metastases were established. The results showed that the expression of PD-L1 correlated well with a Ki-67 value ≥3% and hypertension, indicating that PD-L1 could be considered a malignant behaviour biomarker for Pheo/PGL (175). A study from Bratslavsky et al (176), described the largest series of clinically advanced Pheo/PGL that was evaluated by comprehensive genomic profiling. Eighty-three clinically advanced PGL and 45 clinically advanced Pheo underwent hybrid-capture-based comprehensive genomic profiling (CGP) using a targeted panel of 324 genes and tumour mutational burden (TMB) and microsatellite instability (MSI) were determined. The most frequent potentially targetable genomic alteration in clinically advanced PGL were in *FGFR1* (7%), *NF1*, *PTEN*, *NF2*, and *CDK4* (2%) and for clinically advanced Pheo in *RET* (9%), *NF1* (11%) and *FGFR1* (7%). Both clinically advanced Pheo and PGL had low median TMB, low PD-L1 expression levels and none had MSI high status. Low PD-L1 expression levels and no MSI high status argue against strong potential for novel immune checkpoint inhibitors (176). The results of the phase II clinical trial of pembrolizumab, a humanized anti-PD-1 monoclonal antibody, in patients with progressive metastatic Pheo/PGL, indicate that this drug has modest anti-neoplastic activity with an acceptable safety profile (177). Very recently, Hadrava Vanova et al. examined PD-L1 and PD-L2 expression in relation to oncogenic drivers in their Pheo/PGL patient cohort to explore whether expression can predict metastatic potential and/or be considered a predictive marker for targeted therapy (178). They found that the expression of PD-L1 was elevated in the Pheo/PGL cohort compared with normal adrenal medulla, whereas PD-L2 was not elevated. Expression of PD-L1 was lower in the pseudohypoxia cluster compared with the sporadic and the kinase signalling subtype cluster, suggesting that sporadic and kinase signalling cluster Pheo/PGL could benefit from PD-1/PD-L1 therapy more than the pseudohypoxia cluster. Within the pseudohypoxia cluster, expression of PD-L1 was significantly lower in both SDHB- and non-SDHB-mutated tumours compared with sporadic tumours. PD-L1 and PD-L2 expression was not linked to metastatic behaviour, however, the presence of Pheo/PGL driver mutation could be a predictive marker for PD-L1-targeted therapy and an important feature for further clinical studies in patients with Pheo/PGL (178).

More studies are necessary to increase the number of patients to understand if the expression of PD-L1 and PD-L2 may be linked to the genetic background, and may be possible a personalized treatment targeting the PD-1/PD-L1 pathway depending on the Pheo/PGL clusters.

## Tumour therapy targeting cellular tumour microenvironment

Around 10–15% of Pheo/PGL will become metastatic and have a poor prognosis with a mortality rate of 37% at 5 years (179). In particular, patient *SDHB* mutated present a higher risk of metastatic disease (40). Unfortunately, there are limited options for these cases, based on metabolic radiotherapy or chemotherapy, with imperfect efficacy (180). Knowledge on Pheo/PGL biology has recently been turned upside down by metabolic reprograming, making it possible to envisage the use of targeted therapies, especially keeping in mind that modulating the TME components is critical to regulating solid tumour survival and proliferation (181). Indeed, crosstalk between the tumour cells and TME cells allows malignant tumour cells to evade the host’s anti-tumour immune response, and thus reprogramming the host’s response is crucial for tumour therapy (182).

Targeted molecular therapies are expected to represent the future in the management of patients affected with metastatic Pheo/PGL (**Table 2**). Among these strategies, anti-angiogenic approaches are thought to be highly promising (183, 184). The rationale for targeting tumour vasculature is first based on the well-established observation that Pheo/PGL are very highly vascularized tumours and, therefore, potentially strongly dependent on angiogenesis-mediated growth and survival (119, 120, 122, 123). Targeting the VEGF pathway is the most commonly used anti-angiogenic strategy in cancer and could be a good candidate for inhibiting angiogenesis in Pheo/PGL (183). Sunitinib inhibits cellular signalling by targeting multiple RTKs. These include all platelet-derived growth factor receptors (PDGF-R) and vascular endothelial growth factor receptors (VEGF-R), cKIT and RET. The data of the SNIPP Study (Study Of Sunitinib In Patients With Recurrent Paraganglioma/Pheochromocytoma; ClinicalTrials.gov, identifier: NCT00843037), a multicentric non randomized phase II trial (50 mg per day,4 weeks on and 4 weeks off) that includes 25 patients reported a disease control (stable disease, SD, or partial response, PR) in 83% of treated patients with a median progression free survival (PFS) of 13.4 months (185, 186). The First International Randomized Study in Malignant Progressive Pheochromocytoma and Paragangliomas (FIRSTMAPPP) (ClinicalTrials.gov, identifier: NCT01371201) has been concluded after 8 years of enrolment. This was a multicentric randomize trial (sunitinib 37.5 mg oral once daily: placebo = 1:1) that included 78 patients (32% *SDHx* mutated). The primary endpoint of the study was to evaluate the PFS at 12 months that resulted 35.9% vs 18.9% with a median of 8.9 and 3.6 months, respectively. Considering the results of this trial, Sunitinib could be consider as the first-line option in patients with progressive metastatic Pheo/PGL (187).

**Table 2:** Summary of the ongoing Clinical Trials acting on different intracellular signalling targets.

Study title (identifier number)	Interventions	Target	Phase of the study	Recruitment status
Sunitinib In Patients With Recurrent Paraganglioma/Pheochromocytoma, SNIPP (NCT00843037)	Sunitinib	VEGF1-2-3 PDGF α-β cKIT RET	Phase II	Active, not recruiting
First International Randomized Study in Malignant Progressive Pheochromocytoma and Paraganglioma, FIRSTMAPPP (NCT01371201)	Sunitinib	VEGF1-2-3 PDGF α-β cKIT RET	Phase II	Completed
Belzutifan/MK-6482 for the Treatment of Advanced Pheochromocytoma/Paraganglioma (PPGL), Pancreatic Neuroendocrine Tumor (pNET), or Von Hippel-Lindau (VHL) Disease-Associated Tumors, MK-6482-015 (NCT04924075)	Belzutifan	HIF2α	Phase II	Recruiting
RAD001 in Pheochromocytoma or Nonfunctioning Carcinoid, PheoCarcRAD001 (NCT01152827)	Everolimus	mTOR	Phase II	Completed
Belzutifan (PT2977, MK-6482) in Combination With Cabozantinib in Patients With Clear Cell Renal Cell Carcinoma (ccRCC), MK- 6482-003 (NCT03634540)	Belzutifan + Cabozantinib	HIF2α VEGF	Phase II	Recruiting
Pembrolizumab in Treating Patients With Rare Tumors That Cannot Be Removed by Surgery or Are Metastatic (NCT02721732)	Pembrolizumab	PDL-1	Phase II	Active, not recruiting

Available at: www.ClinicalTrial.gov.

Pheo and PGL included in cluster-1 are characterized by the activation of pseudohypoxic pathways which determines the stabilization of HIF2α, leading to upregulation of VEGF and tumour growth. A new phase II single arm trial with the anti HIF2α Belzutifan (120 mg oral once daily) is ongoing (ClinicalTrials.gov, identifier: NCT04924075). Patients affected by Advanced Pheo/PGL, Pancreatic Neuroendocrine Tumour (pNET), or Von Hippel-Lindau (VHL) Disease-Associated Tumours could be included. The primary endpoint is to evaluate the objective response rate (ORR).

While in cluster-1 patients TKI inhibitors and anti HIF2α inhibitors have to be considered, in cluster-2 patients mTOR inhibitors have a central role due to the activation of MAPK and mTOR signalling pathways. A phase II study on everolimus has been recently concluded. Patients with non-functioning neuroendocrine tumours or Pheo/PGL were treated with everolimus monotherapy (10 mg daily po medication). This trial showed a PFS of 3.8 months in a phase II trial enrolling patients affected by progressive NETs or Pheo/PGLs. Only considering the seven Pheo/PGL patients, 5 presented SD and 2 developed progressive disease (PD), demonstrating a modest efficacy in patients with Pheo/PGL (ClinicalTrials.gov, identifier: NCT01152827) (188).

The possible combination of TKI plus anti HIF2α inhibitor (ClinicalTrials.gov, identifier: NCT03634540) or mTOR inhibitor (186) has to be evaluated. The patients will be divided into 2 cohorts. Cohort1: participants will receive 120 mg belzutifan and 60 mg cabozantinib orally once daily (QD) at the same time. Cohort2: participants who have received prior immunotherapy will receive 120 mg belzutifan and 60 mg cabozantinib orally QD at the same time.

Currently, the results of immune checkpoint inhibitors in Pheo PGL are still controversial. The programmed death 1 (PD-1)/programmed death ligand 1 (PD-L1) pathway is modulated by cancer cells determining immunosuppression leading to tumour growth. A phase II trial with Pembrolizumab, a PD-1 monoclonal antibody, demonstrated the absence of PD after 27 weeks of therapy in four of ten patients with a median PFS of 5.7 months and a median overall survival (OS) of 9 months (ClinicalTrials.gov, identifier: NCT02721732). Patients received pembrolizumab IV over 30 minutes on day 1. Treatment repeated every 21 days for up to 24 months in the absence of disease progression or toxicity. Patients with clinical response or disease stabilization may continue treatment for up to an additional 12 months (177).

## Conclusions

The last decade has seen a growing understanding of the promoting role of TME in cancer progression and spread. TME cells produce several growth factors and cytokines that contribute to establish a close crosstalk with tumour cells. This contributes to the survival of cancer cells, the development of angiogenesis and resistance to therapies. Furthermore, immunosuppressive mediators released by immune cells within the tumour extinguish host-mediated antitumour responses and facilitate tumour progression. Among the various clinical trials that have employed the use of new molecules for the treatment of metastatic Pheo/PGL, some are ongoing and among those concluded sunitinib is the drug with the greatest degree of efficacy, becoming the first-line option in patients with progressive metastatic Pheo/PGL.

Therefore, Pheo/PGL TME as a key driver of tumour progression is considered a new and good candidate for the development of promising drug targets for clinical practice.

## Author contributions

Conceptualization, SM and ER. Writing - draft preparation, SM, FA, LC. Review and editing, ER, SM. Visualization, ER, MM, LC, FA, SM. Supervision, ER. Project administration, E.R. Funding acquisition, ER, MM. All authors contributed to the article and approved the submitted version.
